# Surface protection against corrosion of Ni turbine blades by electrophoretic deposition of MnO_2_, TiO_2_ and TiO_2_–C nanocoating

**DOI:** 10.1039/d2ra06949k

**Published:** 2022-11-24

**Authors:** Qahtan. A. Yousif, Mohammad N. Majeed, Mahmoud A. Bedair

**Affiliations:** University of Al-Qadisiyah, College of Engineering, Department of Materials Engineering Iraq qahtan.adnan@qu.edu.iq; Electric Power Generation Department of Kufa Cement Plant/Processing and Laboratory Research Iraq; College of Science and Arts, University of Bisha P.O. Box 101 Al-Namas 61977 Saudi Arabia bedair@ub.edu.sa; Department of Chemistry, Faculty of Science (Men's Campus), Al-Azhar University Nasr City 11884 Cairo Egypt m_bedier@azhar.edu.eg m_bedier@yahoo.com

## Abstract

The turbine blades of turbochargers are corroded after being cleaned with water in the presence of gasses produced during the combustion of heavy fuel. For that, manganese oxide (MnO_2_), titanium dioxide (TiO_2_), and titanium oxide–graphene (TiO_2_–C) nanomaterials have been coated on the nickel alloy, which is the composition of turbine blades, by the electrophoretic deposition technique for protection against the corrosion process. The anticorrosion performance of nanomaterial coatings has been investigated using electrochemical methods such as open circuit potential, potentiodynamic, electrochemical impedance, and linear polarization resistance in a 1 M H_2_SO_4_ solution saturated with carbon dioxide. The corrosion rate of nanomaterial-coated Ni-alloy was lower than bare alloy, and potential corrosion increased from −0.486 V for uncoated Ni-alloy to −0.252 V *versus* saturated calomel electrode for nanomaterial coated Ni-alloy electrodes. Electrochemical measurements show that TiO_2_ coated Ni-alloy corrosion has good protective qualities, with an efficiency of 99.91% at 0.146 mA cm^2^ current density in sulfuric acid media. The findings of this study clearly show that TiO_2_ has a high potential to prevent nickel alloy turbine blades from corrosion in acidic media. Furthermore, the surface morphologies have revealed that TiO_2_ and MnO_2_ coatings might successfully block an acid assault due to the high adhesion of the protective layer on the nickel alloy surface. The use of X-ray diffraction (XRD) enhanced the various measures used to determine and study the composition of the alloy surface's protective coating.

## Abstract 2

MnO_2_, TiO_2_ and TiO_2_–C nanomaterials have been coated on the nickel alloy, which is the composition of turbine blades, by the electrophoretic deposition technique for protection against the corrosion process.

## Graphical abstract

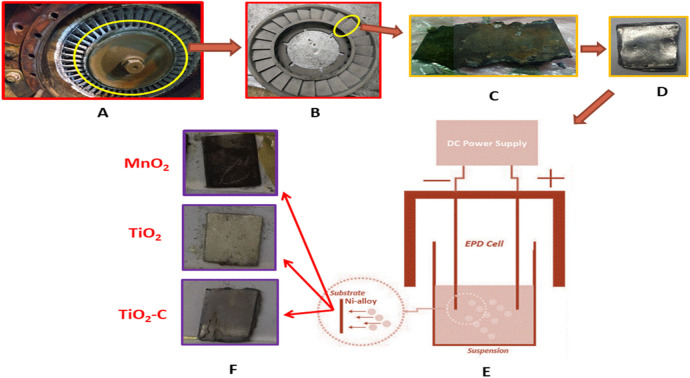

## 1 Introduction

Corrosion is a fundamental phenomenon that plays a critical role in any country's economic structure and security.^1,2^ As a result, it becomes critical to treat different alloys separately.^3^ Additionally, corrosion of steel and nickel alloys is a significant aspect of the industrial concern that has received much attention.^4,5^ Steel and nickel alloys are commonly used in industrial settings, although corrosive in acidic environments.^6–8^ In this period of contemporary civilization, it is critical to avoid unanticipated metal loss by corrosion during the design or operation phases.^9^ Nickel alloys are well suited for soft magnetic applications due to their high permeability and low coercivity.^10^ Nickel and iron alloys are easily corroded when exposed to various acidic media, these acidic solutions are consumed during various production processes.^11^ Therefore, ensuring that nickel alloys maintain their corrosion resistance is a valuable and time-consuming job responsibility.^12^ Numerous techniques are employed to protect the metal surface from destructive attacks (anticorrosive approach); nevertheless, the employment of corrosion inhibitors and surface modification are seen to be practically effective methods of protecting the metal surface, particularly in an acidic medium.^13–15^ In industrial sectors, anions such as sulphates, nitrates, chlorides, and thiosulphates cause corrosion damage to iron alloys, considerably reducing their operating life.^16,17^ Among the different options available for corrosion protection, the most effective method is to use inhibitors and coatings.^18,19^ The specimen surface's protective properties rely on several elements, including the inhibitor–adsorbate interaction, inhibitor incorporation onto the specimen surface, inhibitor concentrations, electrode voltage, temperature, and the corroding specimen surface features.^20,21^ It is well recognized that steady-state and active electrochemical techniques and solution analytical methodologies can be employed to assist in the development of potential corrosion mitigation studies.^22–25^ Insufficient protection against long-haul consumption is provided by the weak bond between the weak oxide layer and the natural covering on the metal surface.^26^ Anodizing in chromic corrosive and plating has been the traditional anti-consumption implementation method.^27^ Nonetheless, growing environmental concerns and stringent restrictions governing the use of chromic corrosive necessitated the development of anodizing electrolytes with a lower environmental impact and associated cleanup costs.^28,29^ Coatings based on oxide nanoparticles are frequently used to protect metal substrates against corrosion under extreme conditions.^30,31^ An electrophoretic deposition technique (EPD) was used to coat the Ni-alloy specimen of the turbine blades with manganese oxide (MnO_2_), titanium dioxide (TiO_2_), and titanium oxide–graphene (TiO_2_–C) nanoparticles.^32^ Unfortunately, the turbine blades corroded when they were cleaned with water in the presence of gasses that yield from the combustion of heavy fuel. It is a cost-effective and environmentally friendly method. The investigation of coating protection was performed on the surface of a nickel alloy in 1 M H_2_SO_4_ solution saturated with carbon dioxide at a fixed temperature (298.15 ± 1 K) using electrochemical methods such as open circuit potential (OCP), potentiodynamic (PD), electrochemical impedance (EIS), and linear polarization resistance (LPR). Field emission scanning electron microscopy (FESEM) and energy-dispersive X-ray spectroscopy (EDS), two surface morphological techniques, were used to assess the nature of the surface coated. X-ray diffraction (XRD) enhanced the other measurements, a crucial non-destructive instrument for determining the alloy surface's protective film composition.

## 2 Experimental part

### 2.1 Preparation of working electrode

**Fig. 1:**
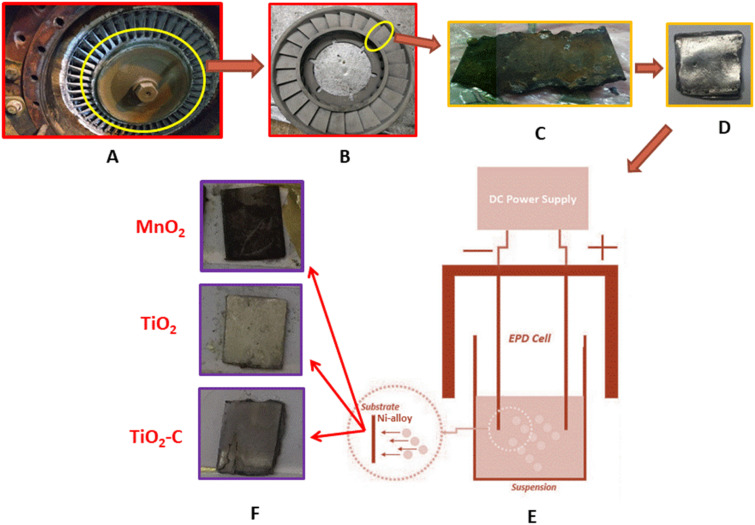
Steps sampling and coating of Ni-alloy (A) turbocharging part, (B) fan blades, (C) cut-up of the specimen, (D) sample polishing, (E) EPD cell, (F) types of coating.

Ni-alloy samples were taken from turbocharging turbine blades for the electrical workstation of the cement plant in the Al-Najaf government, Iraq and contained the following element compositions, as listed in Table 1. Metals analysis is highly trustworthy by the SPECTROMAXx, SPECTRO Analytical Instruments GmbH Company, Germany. An appropriate sample with the following dimensions (1 cm × 1 cm) and thickness was created (0.35 cm), as shown in Fig. 1. All specimens were polished like mirrors with emery sheets of various sizes, and then a soft cloth soaked in diamond paste was employed as a lubricant on the surface of each.

**Table 1:** Composition of Ni-alloy sample

Elements	Mn	C	Al	Ni	Mo	Cr	Si	Fe
Weight percentage	0.12	0.05	0.18	78.40	0.03	15.30	2.26	3.21

The working Ni-alloy was coated with epoxy resin to allow the exposed area of 1 cm^2^ from coming into contact with an electrolyte solution. The protective effects of the coating through metal oxide nanoparticles (supplied by Sigma-Aldrich Company) on the Ni-alloy electrode surface were studied using an electrolyte solution of 1 molar sulfuric acid (also supplied by Sigma-Aldrich Company) in deionized water for 2 hours. The tests were done at 298.15 ± 1 K.

### 2.2 Electrophoretic deposition method

The electrophoretic deposition (EPD) of a well-dispersed MnO_2_ layer on Ni-alloy was carried out utilizing an electrochemical cell consisting of a Nickel alloy (Cathode, 1 cm × 1 cm) sandwiched between two parallel titanium metal electrodes (counter electrodes), as illustrated in Fig. 1. The cathode and counter electrode were separated by 0.5 cm. The MnO_2_ solution was ultrasonicated for about 1 hour before each deposition to ensure that the nanoparticles were dispersed uniformly. EPD was done using a constant DC voltage mode (30 V), a time of 1 minute, and 1 g of nanoparticles in a solvent combination of acetone and absolute ethanol (2 : 1) with the addition of aluminum nitrate nonahydrate (0.1 g.) as a molecular charger. The samples were dried in the air at room temperature for 24 hours and stored in a desiccator until further measurements were performed.

The same procedure of MnO_2_ deposition was used to deposit TiO_2_ and TiO_2_–C onto Ni-alloy, except the composition of EPD and the potential values were changed. In a solution of 30 mL isopropanol and 15 mL ethanol, 0.2 g. TiO_2_ and TiO_2_–C nanoparticles were added which have been prepared in previous works.^33^ After adding 0.03 g. iodine, 10 mL acetone, and 5 mL acetylacetone, the mixture was ultrasonically treated for one hour. The applied voltages were 100 V, 150 V for 1.5 minutes, and 2.5 minutes, respectively, for TiO_2_ and TiO_2_–C.

### 2.3 Electrochemical measurements

A corrosion cell was built utilizing Ni-alloy as the working electrode to examine the protective effects of metal oxide nanoparticles against Ni-alloy corrosion when exposed to carbon dioxide sulfuric acidic solution (1 M) at 298.15 ± 1 K. The electrodes for the reference and auxiliary electrodes were put together. They were placed in a corrosion electrochemical cell simultaneously and connected to the Gamry potentiostat device. At the polarized Ni-alloy working electrode, the device is used to measure the polarization curves and estimate corrosion process parameters such as corrosion potential (*E*_corr_), corrosion current density (*i*_corr_), and anodic and cathodic Tafel slopes (*β*_c_, *β*_a_), and corrosion rate. After achieving a steady state of OCP, electrochemical impedance was measured. An AC signal with a peak-to-peak amplitude of 10 mV was used with a frequency range of 10 kHz to 0.01 Hz in the research at the corrosion potential (−*E*_corr_) to determine full electrochemical impedance parameters and calculate the coating protection percentage.

### 2.4 Characterization of a protective layer

FESEM (ZEISS Gemini, Germany LTD Company) was used to characterize Ni-alloy specimens. First, we recorded the Ni-alloy sample (exposed area ∼1 cm^2^) with and without a coating layer on the electrode surface. FESEM images were acquired to investigate the surface morphology of Ni-alloy. The EDS spectra were analyzed to determine the chemical components' compositions. Before recording each image, the samples were cleaned with deionized water and dried for one hour in a desiccator. The XRD patterns were recorded with the aid of a Bruker D6 Advance diffraction machine fitted with a Cu radiation source (*λ* = 0.1541 nm). Diffraction measurements were taken at two different angles ranging from 5° to 80°.

## 3 Results and discussion

### 3.1 X-ray diffraction (XRD) analysis

**Fig. 2:**
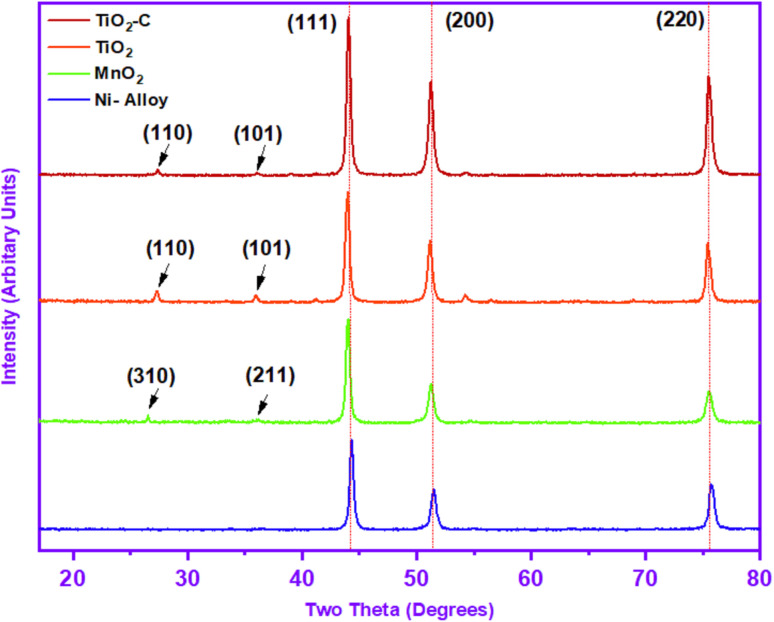
X-ray diffraction pattern of Ni-alloy surface and coating nanomaterials (MnO_2_, TiO_2_, and TiO_2_–C).

X-ray diffraction peaks were measured and reported in Table 2 to identify the crystalline phases of protective coating. The diffractions of (111), (200) and (220) Miller planes may be indexed to the peaks at 44.32°, 51.47° and 75.72°, respectively, as shown in Fig. 2. Due to the high solubility of Cr atoms in the Ni matrix, the pattern suggests that the material is made of Ni at a high intensity.

**Table 2:** XRD analysis peaks of Ni-alloy surface and coating nanomaterials (MnO_2_, TiO_2_, and TiO_2_–C)

Peak Position (2−*θ*)	FWHM (*β*)	Theta(*θ*)	Intensity	Crystallite Size *D* (nm)	Miller index
44.325	0.436	22.162	1164	10.04	111
51.475	0.600	25.737	532	7.504	200
75.725	0.582	37.862	568	8.827	220

**MnO**_**2**_					
26.525	0.332	13.262	124	12.55	310
36.025	0.512	18.012	73	8.330	211
43.975	0.435	21.987	1330	10.05	111
51.225	0.591	25.612	517	7.610	200
75.475	0.688	37.737	432	7.455	220

**TiO**_**2**_					
27.275	0.339	13.637	168	12.31	110
35.925	0.335	17.962	114	12.72	101
43.975	0.461	21.962	1428	9.486	111
51.175	0.535	25.587	817	8.405	200
75.425	0.530	37.715	784	9.674	220

**TiO**_**2**_ **–C**					
27.375	0.424	13.687	103	9.845	110
36.075	0.329	18.037	63	12.96	101
44.025	0.438	22.015	2053	9.988	111
51.225	0.535	25.615	1222	8.407	200
75.475	0.546	37.737	1296	9.393	220

The XRD patterns of Ni-alloy surface and MnO_2_, TiO_2_, and TiO_2_–C nanomaterials coating are shown in Fig. 2. Two of the diffraction peaks in the XRD pattern of MnO_2_ nanoparticles are indexed to the MnO_2_, which is compatible with the JCPDS card (JCPDS-44-0141).^34^ The interlayer reflection at 26.52° (310) and the asymmetric in-plane^35^ reflection at 36.02° (211) in the MnO_2_ XRD pattern imply a tetragonal crystalline structure, exhibiting effective coating on the nickel alloy in the two-dimensional structure -MnO_2_.^36^ The weakness of the diffraction peaks and the absence of any diffraction peak matching to any contaminant also validated the MnO_2_ nanoparticles' thin crystallinity and purity. Furthermore, strong diffraction peaks attributable to the Ni-alloy were seen at 43.97°, 51.25°, and 75.47°, as presented in Table 2.

The corresponding XRD pattern in Fig. 2 revealed that the protective coated TiO_2_ layer on Ni-alloy had polymorphs, with principal peaks at 27.27° and 35.92° attributed to the (110) and (101) rutile diffractions, respectively, as well as the (111), (200), and (220) of Miller index, which consisted of 2-theta at 43.97°,51.17°, and 75.42°, respectively, as shown in Table 2. It is consistent with the conventional XRD diffraction patterns of rutile in the JCPDS dataset (No. 21-1276).^4^ The XRD pattern (Fig. 2) of TiO_2_–C shows the same diffraction peaks for the TiO_2_ at the slight change in the 2-theta position, as displayed in Table 2. A clear peak for the graphene nanoplatelets has not appeared due to the interference with the rutile peak, which is expected to appear at 26.50°, which has a structure between the amorphous and the crystalline phase. Scherrer's equation^37^ was used to determine the average particle size. MnO_2_, TiO_2_, and TiO_2_–C nanoparticles have particle sizes of 11.49 nm, 10.51 nm, and 10.11 nm, respectively. The findings revealed differences in the particle sizes of the protective layer created on the Ni-alloy surface using the electrophoretic technique.

### 3.2 Open circuit potential (OCP)

**Fig. 3:**
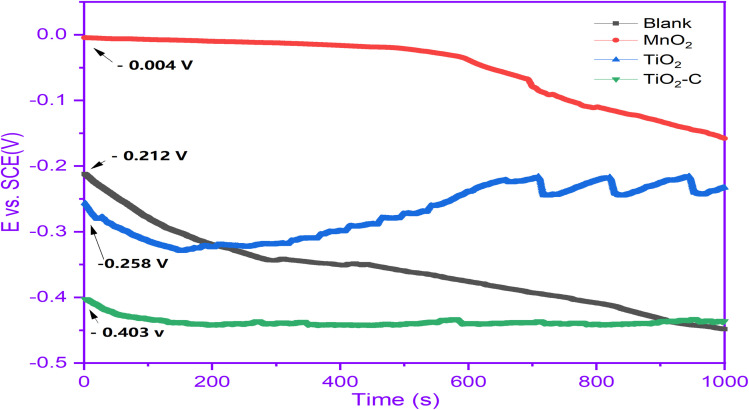
OCP curves without and with coating nanomaterials MnO_2_, TiO_2_, and TiO_2_–C on the Ni-alloy surface.

The variation in OCP as a function of immersion time can characterize the Ni-alloy coating. Fig. 3 illustrates the evolution of the open circuit potential with time for Ni-alloy in 1 M H_2_SO_4_ solution without and with coated protection. The curves demonstrate significant changes in the OCP's temporal behavior due to the nanomaterials protective layer. Without protection, we detect a cathodic displacement of Ni-alloy. However, it is worth noting that with TiO_2_–C, the potential change in the cathodic (active) direction is more pronounced. The drop in OCP is more significant in TiO_2_–C than unprotected, most likely due to the protective layer's adequate isolation of the Ni-alloy surface from the corrosive environment. The profiles of the OCP curves with MnO_2_ and TiO_2_ coatings exhibit typical anodic tendencies to more positive and noble potential.^38^ In the former case (TiO_2_), the achieved potential values were consistently more negative (cathodic) than the uncoated values. However, in the latter case (MnO_2_), the OCP values were continually more positive until the 700 s. This might show that different types of protective layers stick to the surface of the alloy.

### 3.3 Potentiodynamic curves (PD)

**Fig. 4:**
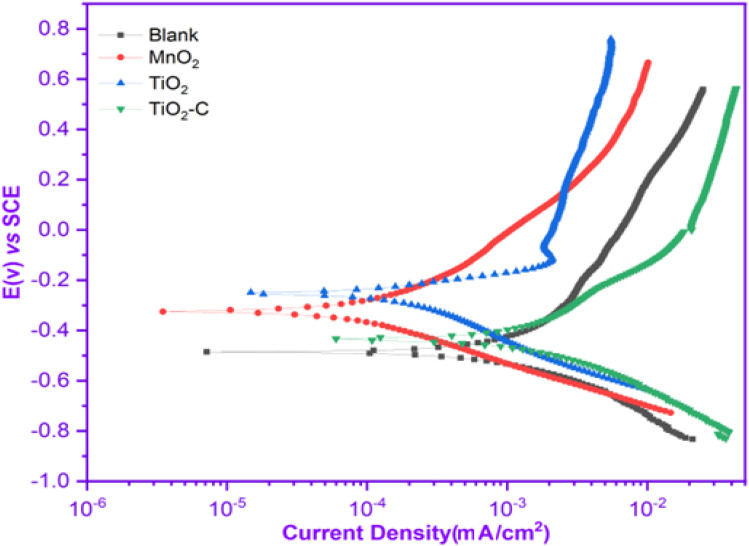
Potentiodynamic polarization curves without and with coating nanomaterials MnO_2_, TiO_2_, and TiO_2_–C on the Ni-alloy surface.

Fig. 4 illustrates the potentiodynamic polarization curves of an uncoated Ni-alloy and MnO_2_, TiO_2_ and TiO_2_–C nanomaterials coated on the Ni-alloy surface. Table 3 contains the corrosion potential (*E*_corr_), current density value (*i*_corr_), anodic Tafel constant (*β*_a_), cathodic Tafel constant (*β*_c_), and corrosion rate (CR) derived from these curves. It was found that the coated samples have lower current density values than the uncoated Ni-alloy. The coated sample's corrosion potential is moved to the anodic direction. This result demonstrates the coating's resistance to corrosive media.^39^ The following equation can be used to calculate the protection efficiency (PE)^40^ based on measurements of corrosion current density.

1 %*η*_pr_ = [(*i*_corr_ − *i*_(coating)corr_)/*i*_corr_] × 100

where *i*_corr_ and *i*_(coating)corr_ are the corrosion current densities values in uncoated and coated, respectively.

**Table 3:** Potentiodynamic polarization parameters without and with coating nanomaterials MnO_2_, TiO_2_, and TiO_2_–C on the Ni-alloy surface

Types of electrocoating	*β*_a_ × 10^−3^ (V per decade)	*β*_c_ × 10^−3^ (V per decade)	*i*_corr_ (mA cm^−2^)	*E*_corr_ (mV *vs.* SCE)	Corrosion rate (mpy)	%*η*_pr_
Blank	682.8	295.5	1.65	−486.0	753.7	—
MnO_2_	341.0	320.6	0.27	−323.0	73.40	83.63
TiO_2_	98.40	190.0	0.12	−252.0	66.58	92.72
TiO2–C	347.8	200.0	0.89	−435.0	675.0	46.06

*i*_corr_ decreased from 1.65 mAcm^2^ for uncoated Ni-alloy to 0.27, 0.12, and 0.89 mAcm^2^ with MnO_2_, TiO_2_, and TiO_2_–C coatings, respectively. The corrosion rate (CR) of MnO_2_, TiO_2_, and TiO_2_–C coated on the Ni-alloy surface are 73.40, 66.58, and 675.0 mpy, respectively, lower than those observed for the uncoated surface. As shown, the corrosion current density of the TiO_2_ coated surface is less than that of the TiO_2_–C coated surface, while the *η*_pr_% is more significant. As a result, it was determined that including graphene nanoplates into the matrix titanium dioxide nanoparticle coatings did not enhance the anticorrosive efficacy of the TiO_2_–C coating on Ni-alloy sufficiently. However, the improved corrosion protection provided by MnO_2_ and TiO_2_ over TiO_2_–C may result from the nanoparticles charged adhering to the electrode surface and protecting it from corrosive substances such as chloride ions, hydrogen, and oxygen gas.

Coating the Ni-alloy surface with a thin film of titanium dioxide and manganese dioxide in 1 M sulfuric acid solution diminished the cathodic and anodic bough slopes, which indicates that hydrogen generation mechanisms were altered by applying a thin film of protection. In other words, it is evident that the hydrogen evolution reaction could be controlled, and the mechanism of the proton discharge reaction varied depending on the protection method.

### 3.4 Electrochemical impedance measurements

**Fig. 5:**
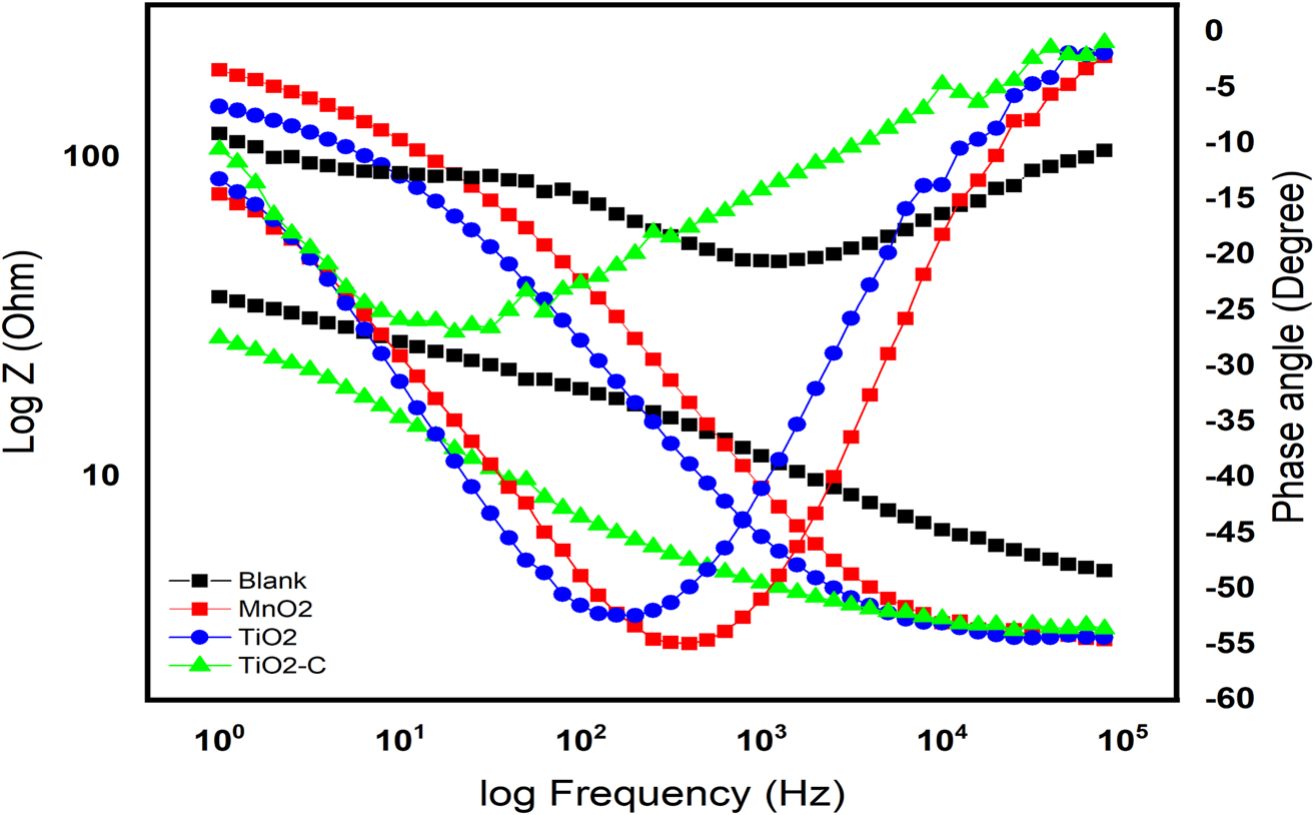
Bode curves without and with coating nanomaterials MnO_2_, TiO_2_, and TiO_2_–C on the Ni-alloy surface.

**Fig. 6:**
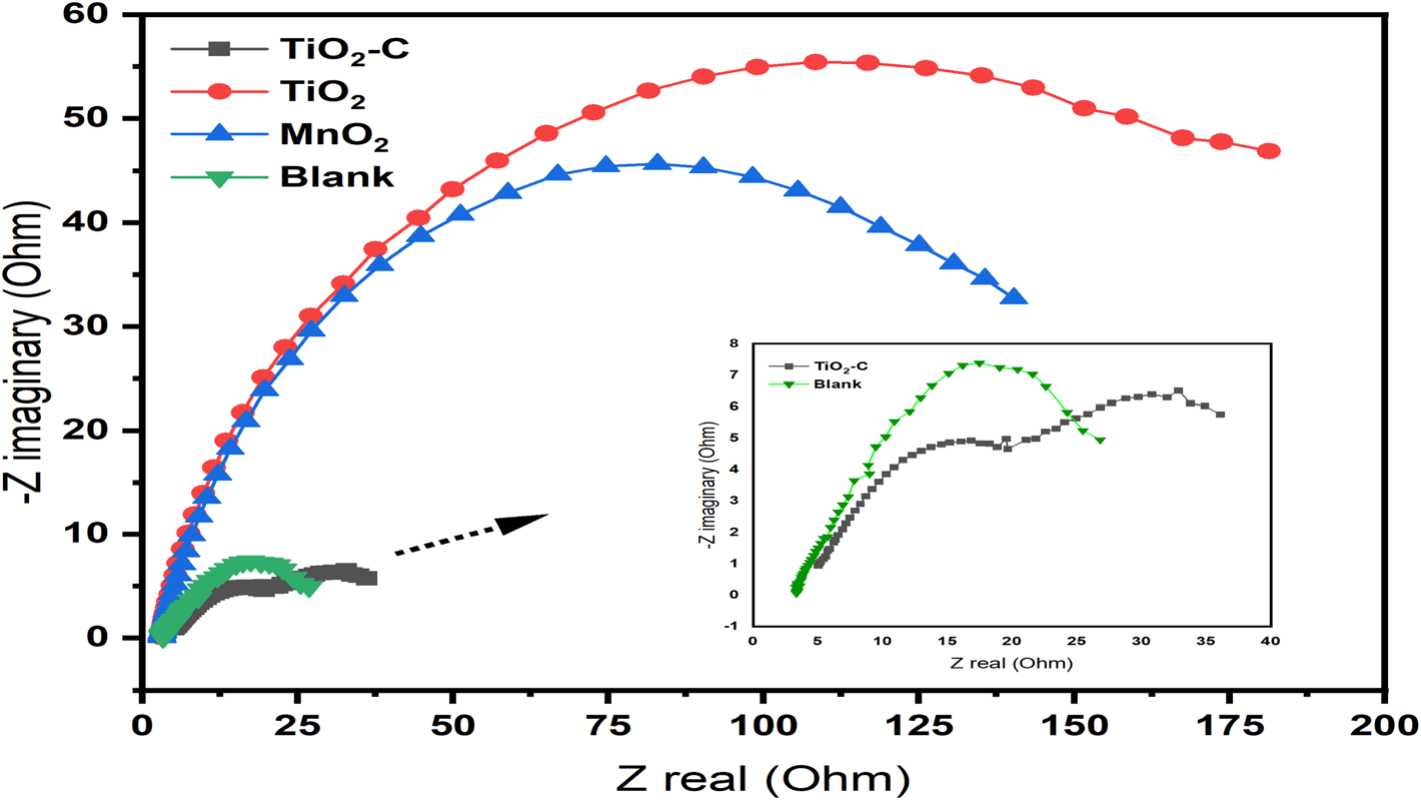
Nyquist curves without and with coating nanomaterials MnO_2_, TiO_2_, and TiO_2_–C on the Ni-alloy surface.

In order to understand the behavior of the Ni-alloy surface electrode, electrochemical impedance measurements were performed. The Bode and Nyquist plots for a Ni-alloy surface immersed in 1 M H_2_SO_4_ and MnO_2_, TiO_2_, TiO_2_–C nanomaterials coated on the Ni-alloy surface are shown in Fig. 5 and 6.

**Fig. 7:**
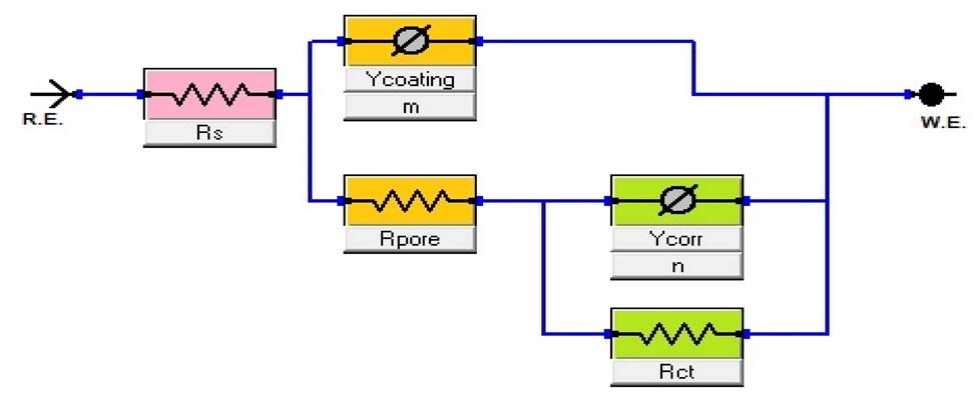
Equivalent circuit model to fit EIS spectra.

The diameter of the semicircle changes and modify as the coating type changes. The variation from the perfect semicircle is generally due to frequency dispersion and the inhomogeneity of the surface, coating grain boundaries, and solution impurities.^41,42^ The equivalent circuit used to fit the electrochemical impedance spectroscopy (EIS) spectra of Ni-alloy in 1 M H_2_SO_4_ with and without the coated layer is shown in Fig. 7.

**Fig. 8:**
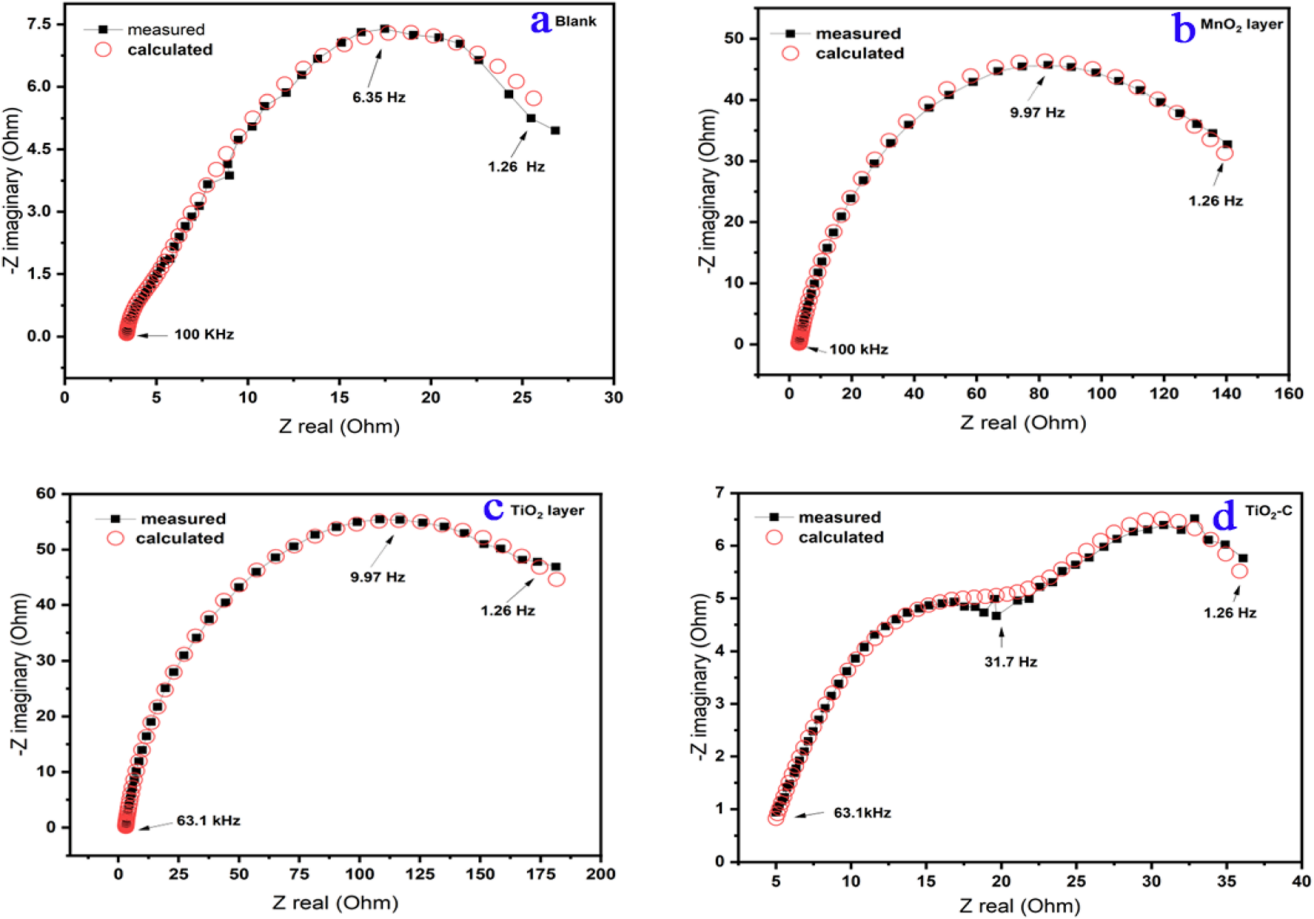
Measurement and calculation fitting curves of Ni-alloy surface and coating nanomaterials MnO_2_, TiO_2_, and TiO_2_–C on the Ni-alloy surface.

The impedance characteristics for the electrolyte “solution” resistance (*R*_s_), the pore resistance (*R*_pore_), and the charge transfer resistance (*R*_ct_) are listed in Table 4. (*Y*_coating_, *m* and *Y*_corr,_*n* are constant phase parameters for the coating layer and corrosion reaction “double layer”, respectively.) Table 4 also includes the double-layer capacitance per unit electrode area (*C*_dl_) and (*C*_dl coating_) that calculated^43^ from the curves depicted in Fig. 8.

**Table 4:** EIS data of coating nanomaterials MnO_2_, TiO_2_, and TiO_2_–C on the Ni-alloy surface

Types of electrocoating/Parameters	Blank	MnO_2_	TiO_2_	TiO_2_–C
*R*_s (_Ω.cm^2^)	4.102	3.088	3.005	3.357
*R*_ct (_Ω.cm^2^)	15.62	172.0	195.6	27.15
*R*_pore_ (Ω.cm^2^)	23.07	20.62	60.40	1.991
*Y*_coating_ (Ω^−1^.s^n^ cm^−2^) ×10^−3^	7.874	1.230	1.277	4.895
*m* × 10^−3^	704.4	326.1	456.3	598.8
*C*_d1coating_ (μFcm^−2^)	0.355	2.960 × 10^−5^	2.006 × 10^−9^	4.389 × 10^−5^
*d* (μm)	—	0.233	0.344	0.157
*Y*_corr_ (Ω^−1^.s^n^ cm^−2^) × 10^−3^	1.226	796.9	89.02	849.5
*n* × 10^−3^	492.5	796.9	826.9	849.5
*C*_d1_ (μFcm^−2^)	3.259 × 10^−3^	4.595 × 10^−4^	2.411 × 10^−5^	1.258 × 10^−3^
*E*_pr_%	—	90.91	92.01	42.46

The following equation was used to determine the percentage protection efficiency^44^ (*E*_pr_%).

2 *E*_pr_% = [(*R*_ct.c_ − *R*_ct_)/*R*_ct.c_] × 100

where *R*_ct,c_ and *R*_ct_ are the charge transfer resistance in with and without coating nanomaterials, respectively.

The data for the *n* exponent (Table 4) range from 0.326 to 0.849, indicating non-ideal capacitance behavior due to the heterogeneity of Ni-alloy surfaces caused by the stiffness of surface coating layer.^45^ The resistances have changed by altering the protective type layer to be the *R*_pore_, and *R*_ct_ are high besides lowering the *R*_s_ value. It indicates the thickness of the protective layer. It lowers the (*C*_dl_) due to an increase in the electrical double layer thickness, indicating that the TiO_2_ nanoparticle introduces the best protective of alloy in optimal condition.

As illustrated in Fig. 5, the Bode diagram indicates that the |*Z*| and phase angles increase as the protective coating layer changes, indicating that it supports a single charge transfer mechanism.^46,47^ As previously stated, an impermeable coating functions as a capacitor; however, with exposure, an inorganic coating deviates from near-perfect capacitor behavior as water, oxygen, and dissolved ionic species permeate the coating to the underlying Ni-alloy surface, resulting in a loss of coating layer effectiveness as TiO_2_–C. The double-layer capacitance lowers when TiO_2_ is coated, indicating that the amount of Ni-alloy elements dissolving has been lowered. Additionally, the coating capacitance value is substantially less, indicating the coating's compact nature. The thickness of the inorganic coatings is essential for industrial applications,^48^ and one of the most important criteria is the thickness of the nanoparticles on the metal. The thickness of coating layers can be measured using EIS data. According to the following equation,^49–51^ the thickness of layers on the surface of Ni-alloy by *C*_dl coating_ can be obtained.

3 *C*_dl coating_ = *ε*_0_*ε A*/*d*

where *ε* the environment's dielectric constant, *ε*_0_ is the vacuum permittivity, *A* is the electrode area, and *d* is the protective layer thickness. As seen in Table 4, the protective efficiency increased as the coating thickness extended. On the other hand, these findings indicate that the barrier qualities of the nickel alloy surface coating enhanced. The order of *E*_pr_ % is TiO_2_ > MnO_2_ > TiO_2_–C, which agrees with the results that obtained from potentiodynamic polarization experiments.

### 3.5 Polarization linear resistance measurements

The corrosion current density is calculated using the well-known linear polarization resistance (LPR) method for determining the corrosion rate. The Stern–Geary equation^52^ was used to determine the polarization resistance (*R*_p_) of Ni-alloy coatings. The coating protection (*η*_LP_%) was calculated using eqn (4)(ref. 53) from the (*R*_p_) values obtained from linear polarization data.

4 *η*_LP_% = [*R*^0^_p_ − *R*_p_/*R*^0^_p_] × 100

where *R*^0^_p_ and *R*_p_ are polarization resistance in the presence and absence of coating layer, respectively.

**Fig. 9:**
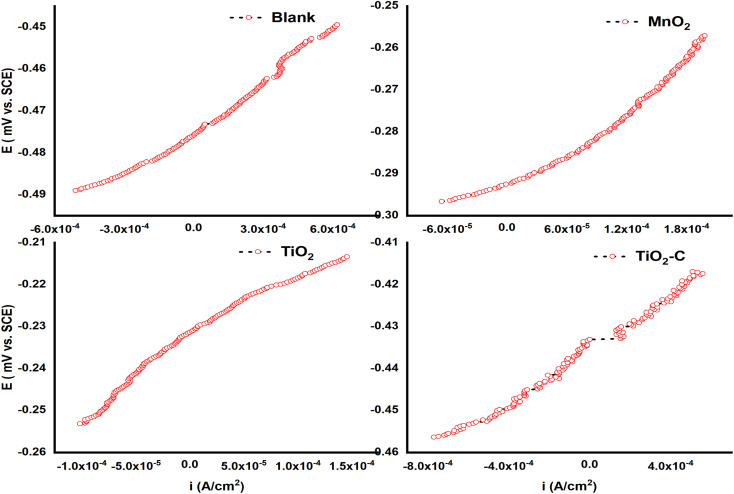
Linear polarization curves of Ni-alloy and coating nanomaterials MnO_2_, TiO_2_, and TiO_2_–C on the Ni-alloy surface.

As illustrated in Fig. 9, typical linear polarization plots of the Ni-alloy and its surface coated with MnO_2_, TiO_2_, and TiO_2_–C nanoparticles were obtained. It is demonstrated that when the protective layer is applied to the surface, the slope of the polarization curves reduces, indicating an increase in polarization resistance, as depicted in Table 5. The polarization resistance (*R*_p_), the corrosion potential (*E*_corr_), the corrosion current density (*i*_corr_), and the corrosion rate are determined using a linear regression calculation on a current density *versus* potential curve near the corrosion potential.

**Table 5:** LRP method information of Ni-alloy and coating nanomaterials MnO_2_, TiO_2_, and TiO_2_–C on the Ni-alloy surface

Types of electrocoating	*i*_corr_ (A cm^−2^)	−*E*_corr_ (mV)	*R*_p_ (Ω cm^2^)	Corrosion rate (mpy)	*η*_LP_%
Blank	1.012 × 10^−3^	433.1	25.74	462.5	—
MnO_2_	320.0 × 10^−6^	292.5	81.42	146.2	68.38
TiO_2_	156.8 × 10^−6^	231.4	166.2	71.63	84.51
TiO_2_–C	712.7 × 10^−6^	475.8	36.55	325.7	29.57

Therefore, the corrosion rate of a nickel-alloy coating is near 71 mpy in a TiO_2_ coating compared to the alloy surface without a coating. According to Table 5, the TiO_2_ coating is more resistant than its Ni-alloy counterpart because the *R*_p_ value is higher (166.2 Ω cm^2^) than that of Ni-alloy (25.74 Ω cm^2^). TiO_2_ and MnO_2_ have the highest *R*_p_ values, but the current density decreases. TiO_2_ coatings have the most substantial polarization resistance and the lowest corrosion rate. Fig. 9 indicates that the corrosion potential of the TiO_2_ and MnO_2_ coatings moves toward more noble values whiles the corrosion potential of TiO_2_–C shifts in the other direction and is more negative. The protection efficiency values derived using potentiodynamic polarization and linear polarization has a different order but is identical. According to certain publications,^54–57^ the total agreement between Tafel extrapolation and linear polarization data is unattainable since the metal surface's morphological structure and roughness varies between anodic and cathodic polarization. Additionally, polarization over a broad potential range does not affect the corrosion system since the corrosion potentials, *E*_corr_ values of the TiO_2_ coating on the Ni-alloy, acquired through linear polarization are comparable to those obtained using potentiodynamic polarization (they differ only by 21 ± mV).

### 3.6 Surface analysis

**Fig. 10:**
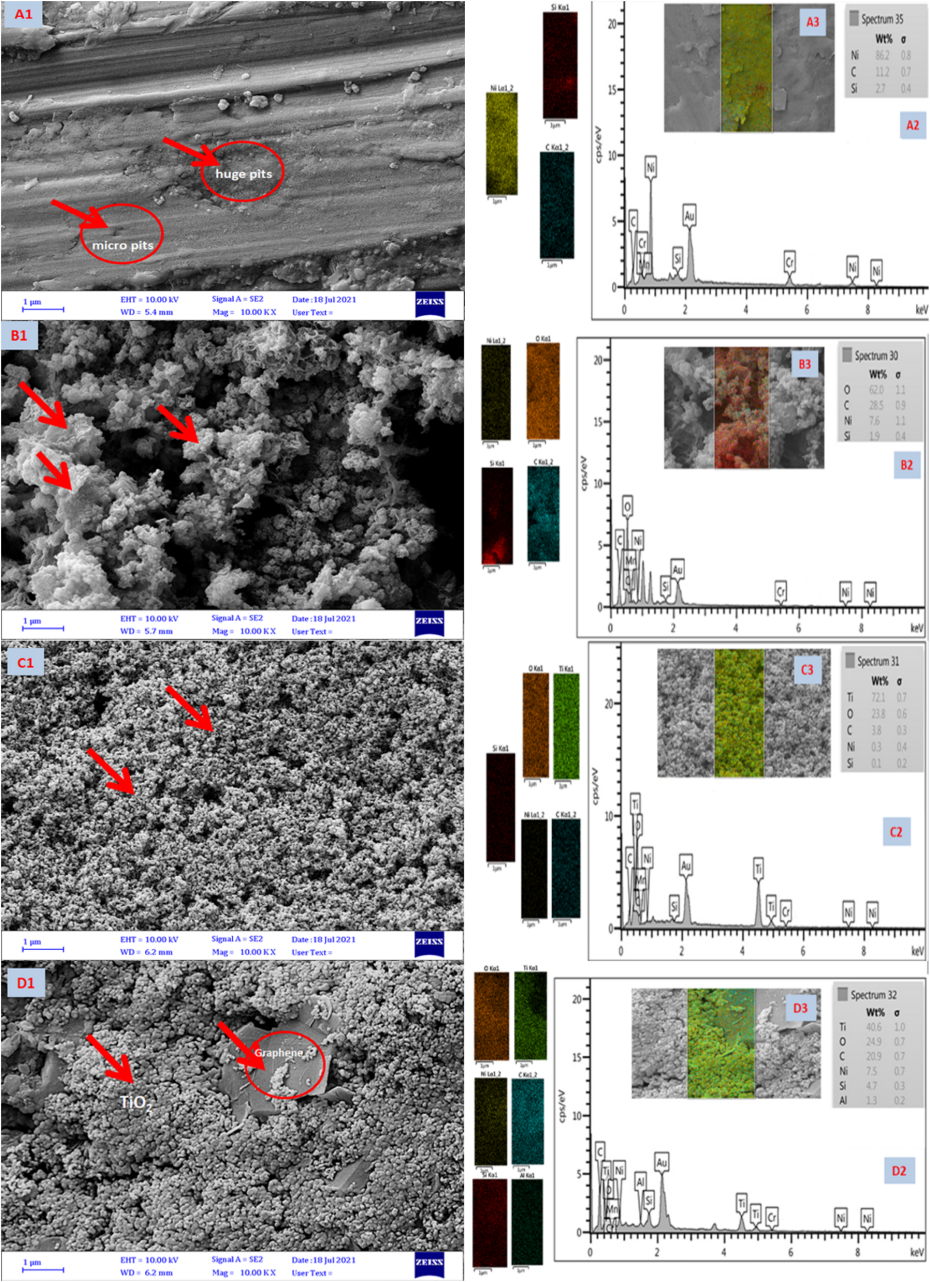
FESEM images (A1, B1, C1, and D1), EDX (A2, B2, C2, and D2), and mapping spectrum (A3, B3, C3, and D3) of Ni-alloy and coating nanomaterials MnO_2_, TiO_2_, and TiO_2_–C on the Ni-alloy surface, respectively.

Fig. 10 shows FESEM images of uncoated Ni-alloy (image A1), TiO_2_, MnO_2_, and TiO_2_–C coatings on the electrode surface of nickel alloy (images B1, C1, and D1). Image A1 demonstrates multiple huge pits and inequalities created due to corrosion, indicating extensive surface damage caused by metal breakdown.^58^ The MnO_2_ coating electrodeposited on the surface protects it from corrosion in image B1, whereas the TiO_2_ coating protects the Ni-alloy in image C. It demonstrates that the coatings created on the nickel alloy surface are -more homogenous and denser. The coatings are of such high quality that no cracks or separation of the coatings are visible. Image D1 demonstrates that the TiO_2_–C coating is brittle and insufficiently cohesive, revealing graphene sheets between the titanium dioxide particles; it is assumed that this resulted in insufficient adherence to the alloy's surface, resulting in low protective efficiency. These findings corroborate electrochemical measurements conduct during the corrosion testing. The EDS elemental analysis is shown in Fig. 10 (A2, B2, C2, and D2) and Table 6. The result indicates that A1 has a higher Ni content. It is lower in the presence of a coating layer from the MnO_2_, TiO_2_, and TiO_2_–C (wt% Ni 7.6, 0.3, and 7.5), respectively.

**Table 6:** Quantitative analysis for of Ni-alloy and coating nanomaterials MnO_2_, TiO_2_, and TiO_2_–C on the Ni-alloy surface from EDX

Element	Ni-alloy	MnO_2_	TiO_2_	TiO_2_–C
	Mass %	Mass %	Mass %	Mass %
Ni	86.2	7.6	0.3	7.5
C	11.1	28.5	3.8	20.9
Si	2.7	1.9	0.1	4.7
O	—	62.0	23.7	24.9
Ti	—	—	72.1	40.6
Al	—	—	—	1.4
Total	100	100	100	100

The EDS of B2 reveals the higher oxygen content from manganese oxide nanoparticles. Besides, the Mn element was not detected by the EDX analysis. The higher Ti content (C2) in the TiO_2_ coating layer on the Ni-alloy. Nevertheless, it is lower in TiO_2_–C to become wt% 40.6, with carbon at 20.9% and oxygen at 24.9%, as shown in Fig. 10, D2. FESEM mapping of elemental distribution as exhibited in Fig. 10 A3, B3, C3, and D3. It is clear to seem the whole surface of Ni-alloy in yellow color is an indicator of homogeneous distribution of the corresponding Ni (A3). Image B3 gave a red color of coated layer for the MnO_2_ nanoparticles. Images of C3 and D3 in Fig. 10 show the distribution of Ti at different weights with oxygen element content as shown in the EDX spectrum (Fig. 10 C3 and D3).

## Conclusion

The results obtained by the experiments described in this work indicate that TiO_2_ is a good coating that was successfully formed on the Ni-alloy specimen by the electrophoretic method, receiving high corrosion resistance in sulfuric acid solution (1 M) saturated with CO_2_. Electrochemical studies showed that the MnO_2_ and TiO_2_ coatings in the corroded acidic solution decrease the values of corrosion current densities and increase the polarization resistance. It is found that the corrosion resistance values are significantly higher for the TiO_2_ and MnO_2_ coatings compared to the TiO_2_–C. The EIS results agree with the potentiodynamic polarization and linear polarization measurements. This study reveals that the TiO_2_ layer has excellent corrosion protection properties and can be considered a potential coating material to protect Ni-alloy against corrosion in a 1 M H_2_SO_4_ solution. We believe that the TiO_2_–C coating did not stabilize the protective film on the nickel substrate enough to stop the corrosion. These observations are supported by FESEM, EDX, and XRD analysis.

## Conflicts of interest

The authors declare that they have no known competing financial interests or personal relationships that could have appeared to influence the work reported in this paper.

## Supplementary Material
